# The natural history of ductal carcinoma in situ: development, validation, and estimated outcomes of the SimDCIS model

**DOI:** 10.1007/s10549-025-07639-0

**Published:** 2025-03-01

**Authors:** Keris Poelhekken, Monique D. Dorrius, Amanda Dibden, Stephen W. Duffy, Bert van der Vegt, Geertruida H. de Bock, Marcel J. W. Greuter

**Affiliations:** 1 Department of Epidemiology, University of Groningen, University Medical Center Groningen, P.O. Box 30 001, FA40, 9700 RB Groningen, The Netherlands; 2 Department of Radiology, University of Groningen, University Medical Center Groningen, PO Box 30.001, EB44, 9700 RB Groningen, The Netherlands; 3 Centre for Cancer Screening, Prevention and Early Diagnosis, Wolfson Institute of Population Health, Queen Mary University of London, Charterhouse Square, London, EC1M 6BQ UK; 4 Department of Pathology and Medical Biology, University of Groningen, University Medical Center Groningen, PO Box 30.001, 9700 RB Groningen, The Netherlands

**Keywords:** Breast neoplasms, Breast ductal carcinoma in situ, Early detection of cancer, Computational modelling, Disease progression

## Abstract

**Purpose:**

To develop a novel simulation model for ductal carcinoma in situ (DCIS), fully validate it, and provide new estimates for DCIS in the setting of population-based biennial screening.

**Methods:**

A micro-simulation Markov model for DCIS (SimDCIS) was developed. Input parameters were independently derived from the literature and transition parameters were age- and grade-dependent. The model was applied to the Dutch biennial screening program. SimDCIS was internally, cross, and externally validated by comparison of the model output to data from the Netherlands Cancer Registry, a modelling study on the United Kingdom Frequency Trial, and the United Kingdom screening program, respectively. Univariate and probabilistic sensitivity analyses were performed to estimate uncertainty. DCIS regression, progression to invasive breast cancer (IBC), clinical detection, and screen-detection were estimated in Dutch screening setting.

**Results:**

SimDCIS matched observed data in internal, external, and cross-validation. The model was most sensitive to DCIS onset probability, and the maximum variation in screen-detection rate was 11%. In Dutch screening setting, DCIS regression, progression to IBC, clinical detection, and screen-detection were estimated at 8% (0–14%), 19% (16–24%), 8% (0–13%), and 61% (56–65%), respectively. Grade distribution was 20% grade 1, 38% grade 2, and 42% grade 3.

**Conclusion:**

SimDCIS provides strong accuracy across validation methods and is particularly sensitive to DCIS onset probability. Most DCIS will be found through screening, of which less than 50% of DCIS will be grade 3, less than 1 in 10 will regress, and 1 out of 5 DCIS will progress to IBC in biennial screening setting.

**Supplementary Information:**

The online version contains supplementary material available at 10.1007/s10549-025-07639-0.

## Introduction

Breast cancer is the most diagnosed cancer worldwide and is often detected at an early stage through population-based screening programs [1, 2]. Ductal carcinoma in situ (DCIS) is considered a stage-zero breast cancer, and its detection rate has steadily increased since the implementation of screening, currently accounting for up to 25% of screen-detected breast cancers and approximately 13% of all breast cancers [3–5]. DCIS can be divided into three grades linked to its malignant potential (1 low malignancy to 3 high) [3, 6, 7]. DCIS is a controversial diagnosis since its increased incidence reflects early detection but can also contribute to overdiagnosis and overtreatment [3, 4, 8]. Overdiagnosis refers to breast cancer that is diagnosed in screening but would not have been diagnosed in a woman's lifetime without screening [3]. Estimates of DCIS overdiagnosis are currently very imprecise and range from 20–91% [3, 6, 9]. These large uncertainties originate from the unknown natural history of DCIS [3, 4, 6]. Large gaps in knowledge exist in the natural history of DCIS due to immediate treatment as standard of care and, consequently, unobservable progression and regression and limited longitudinal data [3, 4]. Current estimates range from 1–10% for regression and 20–91% for progression to invasive breast cancer (IBC) [6]. The unknown natural history of DCIS, therefore, complicates optimization of screening and treatment.

Randomized controlled trials (RCTs) have been set up to explore active surveillance, of grade 1 and 2 DCIS, as an alternative to immediate treatment [3, 4]. These active surveillance trials might provide information about the natural history of DCIS [4]. However, inclusion criteria are strict, and recruitment is slow, so results will take a long time (at least 3 more years) and will only provide a highly restricted image of the natural history of DCIS [3, 4]. Simulation models provide an excellent alternative, as they can give insight into the natural history of DCIS based on observational data, allow for faster and accurate estimates, and assess potential benefits and harms of screening scenarios [6, 10, 11]. Previous DCIS models have reported varying conclusions due to the large range in modelling assumptions [4, 6, 7, 11–16]. To accurately model the natural history of DCIS, progression should vary with the established risk factors age and DCIS grade, but only three models that were previously identified included both [6]. In addition, modelling studies should accurately report assumptions, provide full validation, and adequately assess uncertainty [17, 18], but previously identified DCIS models have shown limited validation and uncertainty assessment [6, 17].

Therefore, the aim of this study was to develop and fully validate a simulation model for DCIS in the setting of population-based breast cancer screening, including age- and grade-dependent progression, to provide more precise estimates of DCIS. Improved estimates of DCIS will help improve the accuracy of overdiagnosis estimates, quantify the influence of important factors such as age and grade, and eventually optimize screening to benefits and harms associated with both IBC and DCIS.

## Methods

### DCIS model

**Fig. 1:**
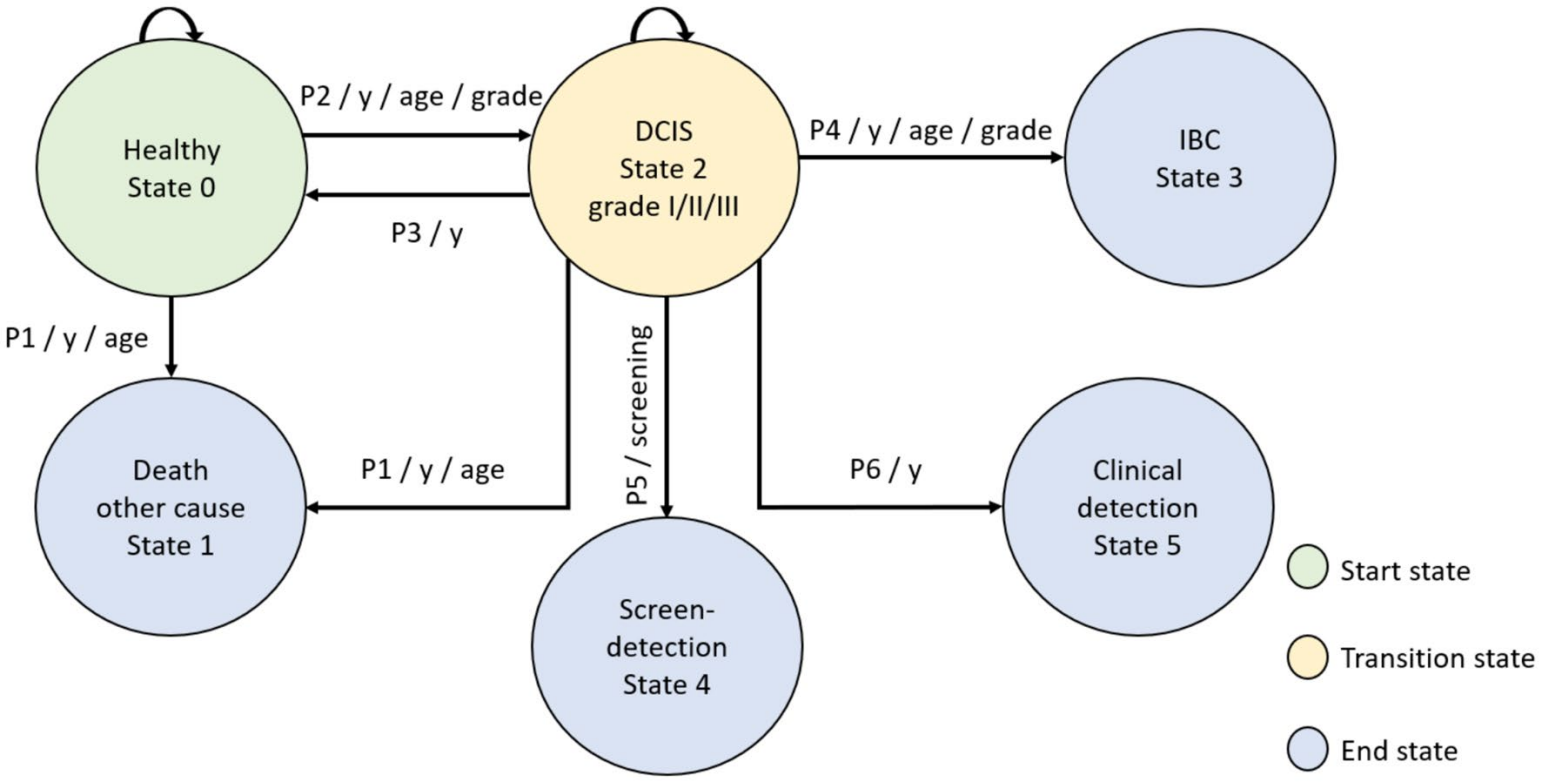
Visual representation of the SimDCIS model. Transition probabilities P1 to P6 per year (y), age, and grade

A micro-simulation Markov model to simulate DCIS, SimDCIS, was developed (Fig. 1). With the model, a virtual cohort of an arbitrarily large number of women can be created to mimic a true population. Within this cohort, each woman is followed yearly from birth until they reach one of the following states: death, IBC, screen-detection of DCIS, or clinical detection of DCIS. At every age, each individual woman has an age-dependent chance to die, and an age-dependent chance to develop DCIS. If a woman develops DCIS, she has a probability to die, remain in DCIS state, progress to IBC, regress to healthy state, or be clinically or screen- detected. The age-dependent death probability in DCIS state is equal to that in the healthy state, as DCIS without progression to IBC does not change the death probability since death of DCIS is extremely rare [6, 16]. Each DCIS grade has its own probability of regression, detection, and grade-dependent probability of progression to IBC. Direct progression from healthy to IBC is assumed to be possible but is outside the scope of this model [6]. Progression from DCIS to IBC made it possible to determine the number of DCIS cases that become invasive, which is important to consider in, for example, overdiagnosis estimations. Regression was only possible for DCIS, where regression of IBC was assumed not possible [6]. The SimDCIS model can be used to simulate different scenarios, where for one scenario, the model output can be averaged over several cohorts.

### Model input parameters

The model input consists of four main transition probabilities (death, DCIS onset, regression of DCIS, progression to IBC), four screening parameters (screening age, frequency, participation, sensitivity), and one clinical detection parameter. All model input parameters were independently derived from existing literature and data (Table 1).

**Table 1:** Input parameters of SimDCIS for the Dutch screening setting

Parameter			Base value			Reference
The Netherlands		*Age (y)*				
Transition	All-cause death probability, P1 (*10^–2^ / year)	0	0.324			[19]
		1–9	0.050 – 0.008			
		10–19	0.008 – 0.018			
		20–29	0.019 – 0.027			
		30–39	0.032 – 0.064			
		40–49	0.070 – 0.201			
		50–59	0.233 – 0.496			
		60–69	0.538 – 1.137			
		70–79	1.210 – 3.441			
		80–89	3.972 – 12.55			
		90–99	14.33 – 32.30			
		100	100.0			
	DCIS onset, P2 (*10^–3^ / year)	*Age (y)*	*Grade*			[5, 29]
			*1*	*2*	*3*	
		0–19	0.0000	0.0000	0.0000	
		20–24	0.0016	0.0003	0.0013	
		25–29	0.0019	0.0043	0.0075	
		30–34	0.0017	0.0127	0.0199	
		35–39	0.0119	0.0221	0.0406	
		40–44	0.0213	0.0516	0.0526	
		45–48	0.0398	0.0600	0.0665	
		49–54	0.2095	0.3105	0.3250	
		55–59	0.0859	0.1805	0.2558	
		60–64	0.0948	0.2396	0.3002	
		65–69	0.1016	0.2674	0.2857	
		70–75	0.1540	0.3556	0.3703	
		76–79	0.0307	0.0630	0.0491	
		80–95	0.0361	0.0585	0.0452	
		95 +	0.0000	0.0000	0.0000	
	DCIS regression, P3 (/year)	20 +	0.0488	0.0488	0.0488	[6, 20, 29]
	Progression to IBC, P4 (/year)	0–19	0.000	0.000	0.000	[21, 29]
		20–54	0.087	0.137	0.159	
		55 +	0.073	0.115	0.134	
Screening	Mammographic sensitivity, P5		86%			[24]
	Screening frequency		Biennial			[23]
	Screening age		50–74 years			
	Participation rate		76%			
Clinical detection (/year), P6			5%			[7]

Input parameters for SimDCIS for the Dutch population. *DCIS* ductal carcinoma in situ, *IBC* invasive breast cancer

#### Transition probabilities

Death probability (P1) was age-dependent and calculated for each year of age from Dutch mortality data from 2011 from the Central Bureau of Statistics (CBS) [19]. The probability to develop DCIS (P2) was determined from the average age- and grade-dependent incidence of DCIS from the Netherlands Cancer Registry (NCR) of 2015–2022, excluding data from 2020 because of data variations due to the COVID-19 pandemic [5]. This DCIS onset probability was calculated for each age and grade. Regression (P3) was included from DCIS to a healthy state and set to 5%, independent of age and grade, as the literature showed that regression was estimated below 10% [6, 20]. If a DCIS regressed and a woman returned to healthy state, probability to transition to DCIS again remained equal to P2. The probability of DCIS progression to IBC (P4) was derived from age-dependent data from the US National Cancer Institute’s Surveillance, Epidemiology, and End Results program of 1992–2014 [21]. Grade dependency was derived from NCR data and assumed equal to grade dependency of DCIS onset [5]. Estimation of transition probabilities is described in detail in Appendix B1.

#### Screening parameters

The model requires the input of four screening parameters: screening age, frequency, participation, and sensitivity. Sensitivity (P5) can be adjusted, so multiple screening detection methods could be studied. For this study, the base input of the model was adjusted to the Dutch screening program (Table 1). The breast cancer screening program was rolled out across the Netherlands between 1989 and 1998 [22]. All women aged 50–75 years without a history of breast cancer are invited for a biennial mammographic examination, which has a DCIS-specific sensitivity of 86% [23, 24]. Screening participation rate was 76% in 2019 [23].

#### Clinical detection

A present DCIS can be either screen- or clinically detected. Clinical detection entails all detections outside of the screening program, e.g. opportunistic or high-risk screening, and symptomatic and accidental detections [7, 25]. The probability of a present DCIS to be clinically detected was 5% per year (P6), independent of age, and DCIS grade [7].

### Model output

The raw model output consists of the yearly state for each individual woman and a summary table (Appendix B2). The summary table includes the number of DCIS per grade, regressed DCIS, women removed from the model per state (death, screen detected, clinically detected, or IBC), and mammograms. In addition, the data can be used to extract the average age of all events and the detection rate per 1,000 screened women stratified by age and grade. Through comparison of multiple scenarios, additional outcomes, such as overdiagnosis rate, can also be calculated. The main model outcome can be determined to fit the research question.

### Validation

To fully validate the model, internal, external, and cross-validation were performed [18]. Internal validation of the model was performed by comparison of simulated 95% confidence intervals (95%CI) with observed NCR data from 2019 (Table 1, Appendix A1) [5]. The outcome used for internal validation was DCIS screen-detection rate (number of screen-detected DCIS per 1,000 screened women) stratified by age and grade. For this comparison, the base scenario in Dutch screening setting was simulated, with biennial screening from age 50 to 75 and 76% screening compliance [23]. External validation was performed by comparison with observed data from the National Health Service (NHS) of 2021 [26]. To simulate the UK screening setting, the base scenario was adjusted to triennial screening, screening age 50–71 years, and UK mortality rates from 2019 (Appendix A2) [26, 27]. For external validation, the main outcome was DCIS screen-detection rate stratified by age. In addition, cross-validation was performed by comparison of the number of DCIS detected in the UK Frequency trial simulated by SimDCIS and MISCAN-FADIA (Appendix A3) [28].

### Sensitivity analyses

To fully analyse the robustness and uncertainty of the model, univariate sensitivity analyses (USA) and a probabilistic sensitivity analysis (PSA) were performed. USA were performed to evaluate robustness of the model. For each input parameter, the lower and upper 95%CI were applied (Appendix A4.1). Changes in DCIS screen- and all-detection rate were recorded overall, stratified by age and grade, and summarized in tornado plots. In addition, a PSA was performed to evaluate the overall model uncertainty through simulation of 100 Monte Carlo scenarios with random selection within the 95%CI of all input parameters (Appendix A5.1). The main outcome of the PSA was screen-detection rate of DCIS per 1,000 screened women.

### DCIS estimates

For each simulated scenario of the PSA (Appendix A6), output was summarized to provide an overview of DCIS estimates within SimDCIS in various circumstances within the setting of population-based screening. Included estimates were percentages of DCIS regression, progression to IBC, screen detection, clinical detection, and the distribution of grades 1, 2, and 3 in screen-detected DCIS.

## Results

### Model validation

For internal validation, the simulated screen-detection rate of SimDCIS showed no significant deviation from observed data for all age groups and grades (Table 2). The simulated grade distribution matched the observed distribution for screen-detected tumours. External validation of the UK screening setting matched NHS data, with only an overestimation of screen-detected DCIS for women aged 50–54 years and an underestimation for women aged 70–74 years (Table 2). Cross-validation showed that SimDCIS matched both study and control groups of the UK Frequency trial. The underestimation of the control group with the MISCAN-Fadia model compared to the trial was not present in SimDCIS (Table 2).

**Table 2:** Validation of SimDCIS

			Screen-detection rate per 1,000 screened women (95% CI)			Reference
			SimDCIS		Observed	
The Netherlands			Internal validation			
Age group	50–54		1.7 (1.5–1.9)		1.6	[5]
	55–59		1.1 (0.9–1.2)		1.1	
	60–64		1.2 (1.0–1.3)		1.0	
	65–69		1.4 (1.3–1.6)		1.5	
	70–74		1.7 (1.5–1.9)		1.9	
Grade	1		0.3 (0.2–0.3)		0.2	
	2		0.5 (0.5–0.6)		0.5	
	3		0.6 (0.5–0.7)		0.6	
Total screen-detected DCIS			1.43 (1.29–1.60)		1.38	
The United Kingdom			External validation			
Age group		50–54	1.9 (1.7–2.1)		1.6	[26]
		55–59	1.5 (1.4–1.7)		1.5	
		60–64	1.5 (1.4–1.7)		1.8	
		65–69	1.7 (1.6–1.9)		1.9	
		70–74	1.9 (1.7–2.1)		2.4	
Total screen-detected DCIS			1.7 (1.6–1.9)		1.7	
UK Frequency Trial			Cross-validation			
Trial arm			SimDCIS	MISCAN-Fadia	Observed	[19, 28]
Study			62 (46–72)	50	52	
Control			45 (30–53)	27	40	

Validation of the screen-detection rate per 1,000 screened women in SimDCIS. Internal validation of the Dutch screening setting stratified by age and grade. External validation of the UK screening setting stratified by age. Cross-validation of the UK Frequency Trial observed and simulated by MISCAN-Fadia

### Sensitivity analyses

In USA, screen-detection rate was most sensitive to DCIS onset probability, with a 10% increase in detection rate at the onset maximum estimate (Appendix A4.2). Probability of regression, progression to IBC, clinical detection, and participation rate showed an inverse effect, with a higher detection rate for lower input estimates. Screen-detection rate per age group and grade showed the same sensitivity as the overall detection rates. The PSA showed an uncertainty of 11% in screen-detection rate (Appendix A5.2).

### DCIS estimates

**Fig. 2:**
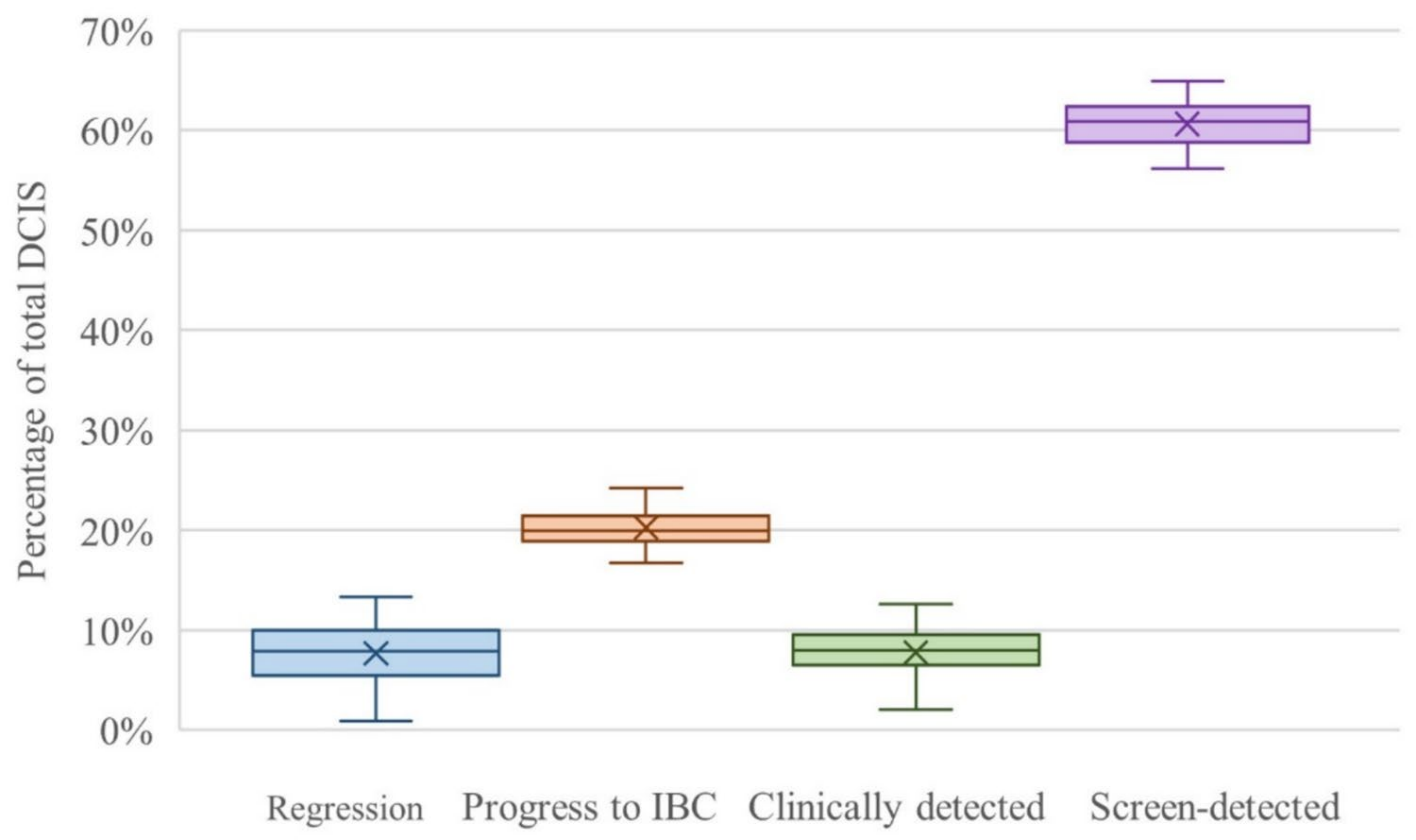
Box-and-whisker plot of the variation in DCIS estimates in SimDCIS as a percentage of total DCIS for the scenarios of the probabilistic sensitivity analysis in the Dutch screening setting

In the Dutch screening setting, the estimated DCIS regression, progression to IBC, clinical detection, and screen-detection were 8%, 19%, 8%, and 61%, respectively (Fig. 2). Regression ranged from 1–14%, but in most scenarios a narrow range of 5–10% was found. Progression to IBC ranged from 17–24%, clinical detection from 2–13%, and screen-detection from 56–65%. The grade distribution, 20%, 38%, and 42% for grades 1, 2, and 3, respectively, showed little change in different scenarios (Appendix A5.2).

## Discussion

The newly developed SimDCIS model was successfully validated, with an excellent match of simulated screen-detected DCIS stratified by age and grade to observed data from Dutch and UK screening. Model uncertainty was highest for a change in DCIS onset probability, with a maximum variation in screen-detection rate of 11%. DCIS estimates for regression, progression to IBC, clinical detection, and screen-detection in the Dutch screening setting were 8% (with a range of 0–14%), 19% (16–24%), 8% (0–13%), and 61% (56–65%), respectively. Screen-detected grade distribution was 20%, 38%, and 42% for grades 1, 2, and 3, respectively.

The SimDCIS model was successfully constructed and validated with accurate simulations of the Dutch and UK screening settings for screen-detected DCIS by age and grade. Internal, external, and cross-validation showed that simulated data matched observed data. Only an overestimation of women aged 50–54 years and an underestimation of 70–74 years in UK screening were observed. Overestimation of 50–54 years of age and underestimation of 70–74 years in the UK setting could be explained by deviations in age at screening in the UK setting. DCIS was detected at screening in women aged 45–49 and 70 + years in observed data as a part of the AgeX trial active in the UK since 2009 to assess the effect of inviting women age 47–49 and 71–73 years [30]. This caused some DCIS to be detected before 50 and after 71 years, which was not possible in the model. Apart from these subtle differences, overall validation was very good. Therefore, SimDCIS was shown to accurately simulate the natural history of DCIS and deemed suitable to provide accurate estimates of DCIS.

New DCIS estimates from SimDCIS were in line with previous literature. Regression of DCIS was previously estimated at 1–10% [6, 20]. SimDCIS showed 8% regression, with a broader range of 0–14%. This range was only broader when the regression input parameter was varied to its boundaries. For all other parameter variations, regression was estimated at 7–8% (Appendix A4.2). SimDCIS showed 16–24% progression of DCIS to IBC suggesting that only approximately 1 in 5 DCIS will progress to IBC. Previous estimates of DCIS progression to IBC ranged from 20–91% [6], but recent literature shows evidence for the lower end of the range. Non-progressive screen-detected breast cancers were estimated at 15.7% in Norwegian screening, with 18% DCIS of total breast cancers as probable largest contributor, which suggests a similar estimate [31]. In addition, a recent review suggested that only 1 in 6 of detected DCIS will progress to IBC [32]. Screen-detected grade distribution was estimated at 20%, 38%, and 42% for grades 1, 2, and 3, which matched Dutch data of 17%, 38%, and 45%, respectively [5]. Previous literature reported 18–20%, 31–32%, and 48–51% for grades 1, 2, and 3 in the Netherlands up to 2015 [7, 33], whereas pathology studies have found 14%, 42%, and 43%, respectively [3]. This might indicate a relatively lower detection of grades 2 and 3 compared to grade 1 in the current Dutch screening setting.

There is consensus that DCIS contributes considerably to overdiagnosis, while on the other hand, increased screen-detection rate of DCIS is associated with lower IBC rates [3, 34]. The extent to which overdiagnosis occurs is difficult to establish given the unobservable natural history of DCIS due to the standard of immediate treatment upon detection [3]. Improved estimation of DCIS natural history parameters and DCIS estimates in screening setting will improve estimations of DCIS overdiagnosis, which currently range from 20–91% [3, 6, 9]. Improved overdiagnosis estimates stratified by established risk factors, such as age and grade, could provide insight into which women could be offered active surveillance instead of treatment, as women with low-risk DCIS offered the choice between conventional treatment and active surveillance showed a preference for the latter [35].

This study has several limitations. First, the model is limited by the availability and quality of existing data. Most data used were recent data from the Netherlands and UK, which are both high-quality databases, but for number of mammograms per age, the most recent data available were from 2011. Also, the UK Frequency Trial took place from 1989 to 1996. As this was the only comparable incidence data used by a DCIS model, it was suitable for cross-validation with adjustment of mortality, screening sensitivity, and participation, as DCIS natural history is not expected to be different over time. Furthermore, DCIS onset probability was derived from observational data, as it is unknown how many DCIS currently remain undetected. However, with current technical standards and relatively low numbers of DCIS, it was assumed negligible. Second, the model only provides a simplification for the general population. Simulations of high-risk women were not included, which suggests a possible underestimation of present DCIS and clinical detection, although the number of DCIS was very small, and model uncertainty is expected to be significantly larger. Currently, age and DCIS grade were considered to stratify onset and progression risk, but other risk factors could be established in future research, which might require additions to the model. In addition, the focus of this study was on screen-detected DCIS. Most diagnoses that contribute to overdiagnosis are the screen-detected tumours, and clinical detection is rare.

Also, several strengths should be considered. SimDCIS includes age- and grade-dependent input parameters, to provide more accurate estimates based on these two well-established risk factors for DCIS onset and progression [6, 7]. Previous DCIS models have been constructed but showed a large range of conclusions [6, 7, 11–16], were based on a large range of assumptions [4, 6], or did not include differences in progression to IBC for varying ages and grades [6]. Furthermore, modelling studies should provide a full validation and sensitivity analysis to ensure that results are valid and reliable and that assumptions are reported to enhance transparency [17, 18]. In this study, internal, external, and cross-validation and univariate and probabilistic sensitivity analyses were performed, which showed valid and reliable results. In addition, SimDCIS only needed ~ 35 seconds to run 1 scenario of 10 iterations with 100,000 women. Therefore, SimDCIS contributes to breast cancer research, because it accurately simulates DCIS natural history, provides fast and accurate estimates, and assesses benefits and harms of different scenarios. Also, DCIS estimates were made in many scenarios and still narrowed existing estimates. Therefore, the estimates from this study can inform on existing gaps in knowledge and contribute to potentially more accurate estimates of overdiagnosis.

## Conclusion

In conclusion, SimDCIS is suitable for accurate benefit and risk estimations of DCIS stratified by age and grade in population-based screening settings and was successfully used to provide accurate estimates of DCIS. DCIS was estimated at 8% regression, 16–24% progression to IBC, and a grade distribution of 20%, 38%, and 42% (grades 1, 2, and 3). Most DCIS will be found through screening. Large gaps in knowledge exist due to the unobservable natural history of DCIS, but information is warranted to accurately quantify overdiagnosis and fuel the discussion on whether or not to treat DCIS upon detection. Results of RCTs will take many years and existing modelling studies show large variations in assumptions and the extent of validation. This showed the need for a well-developed, fully validated model that takes age and DCIS grades into account. In future research, SimDCIS could be used to estimate DCIS overdiagnosis, which currently ranges from 20–91%, to study the influence of age, grade, and different screening scenarios, and to optimize screening guidelines.

## Supplementary Information

Below is the link to the electronic supplementary material.

Supplementary file1 (PDF 1118 KB)
One additional file is available: “Supplementary file – Appendix A and B.docx”. This file contains Appendix A with supplementary tables & figures, and Appendix B with additional information on SimDCIS.

## Data Availability

The SimDCIS model used in this article is publicly available in GitHub at 10.5281/zenodo.11045572 or https://github.com/kp-gith/SimDCIS, in programming language C + + . The dataset supporting the conclusions of this article regarding the Dutch screening can be requested for the purpose of research or statistics from NCR [5]. All other datasets supporting conclusions of this article are publicly available online [19,22,23,26,27].

## Abbreviations

CBS: Central Bureau of Statistics
CI: Confidence interval
DCIS: Ductal carcinoma in situ
IBC: Invasive breast cancer
NCR: Netherlands Cancer Registry
NHS: National Health Service
NL: The Netherlands
PSA: Probabilistic sensitivity analysis
UK: The United Kingdom
USA: Univariate sensitivity analysis
