# Supplementary material for: The natural history of ductal carcinoma in situ: development, validation, and estimated outcomes of the SimDCIS model
Source: Breast Cancer Res Treat. 2025 Mar 1;211(1):223–31. doi: 10.1007/s10549-025-07639-0 (PMC11953075; doi:10.1007/s10549-025-07639-0)
Supplement: Supplementary file 1 — Supplementary file1 (PDF 1118 KB) One additional file is available: “Supplementary file – Appendix A and B.docx”. This file contains Appendix A with supplementary tables & figures, and Appendix B with additional information on SimDCIS. [file 10549_2025_7639_MOESM1_ESM.pdf]

Supplementary files of "The natural history of ductal carcinoma in situ: development, validation, and estimated outcomes of the SimDCIS model" in Breast Cancer Research and Treatment

Keris Poelhekken[a,b], MSc; Monique D. Dorrius[b], MD, PhD; Amanda Dibden[c], MSc; Stephen W. Duffy[c], prof; Bert van der Vegt[d], PhD; Geertruida H. de Bock[a], prof; Marcel J.W. Greuter[b], PhD.

[a] University of Groningen, University Medical Center Groningen, Groningen
Department of Epidemiology
P.O. Box 30 001, FA40, 9700 RB, Groningen, The Netherlands

[b] University of Groningen, University Medical Center Groningen, Groningen
Department of Radiology
PO Box 30.001, EB44, 9700 RB, Groningen, The Netherlands

[c] Queen Mary University of London
Centre for Cancer Screening, Prevention and Early Diagnosis, Wolfson Institute of Population Health
Charterhouse Square, London EC1M 6BQ, United Kingdom

[d] University of Groningen, University Medical Center Groningen, Groningen
Department of Pathology and Medical Biology
PO Box 30.001, 9700 RB, Groningen, The Netherlands

* Correspondence: k.poelhekken@umcg.nl, [a]

Content

Appendix A – Supplementary tables & figures 3

A.1. Validation - Internal 3

A.1.1. Validation – Internal - Description *3*

A.1.2. Validation – Internal - Input *4*

A.2. Validation - External 5

A.2.1. Validation – External - Description *5*

A.2.2. Validation - Input - External *5*

A.3. Validation - Cross 6

A.3.1. Validation – Cross – Description *6*

A.3.2. Validation – Cross - Input *7*

A.4. Univariate sensitivity analyses 8

A.4.1. Univariate sensitivity analyses - Input *8*

A.4.2. Univariate sensitivity analyses *9*

A.5. Probabilistic sensitivity analysis 11

A.5.1. Probabilistic sensitivity analysis – Input *11*

A.5.2. Probabilistic sensitivity analyses – Output *12*

A.6. Overview scenarios output 13

Appendix B – SimDCIS 14

B.1. Calculation transition probabilities 14

B.1.1. P1 - Death probability *14*

B.1.2. P2 - DCIS onset probability *14*

B.1.3. P3 – DCIS regression probability *15*

B.1.4. P4 – DCIS progression to IBC probability *15*

B.2. Model output 16

B.2.1. Model output - Codebook *16*

B.2.2. Model output – Example output *17*

References 18

Appendix A – Supplementary tables & figures

A.1. Validation - Internal

A.1.1. Validation – Internal - Description

Internal validation was done by comparison of observed Netherlands Cancer Registry (NCR) data of the Dutch screening in 2019 to simulated output of SimDCIS (1). For this simulation, the Dutch cancer screening setting was mimicked. Input parameters were determined as close to the Dutch population as possible (Appendix A1.2). During construction of the model, best estimates were collected for the probability of DCIS onset, regression, and progression to invasive breast cancer (Appendix A1.2, Appendix B1). Raw output of the simulation was given as number of DCIS and number of mammograms (Appendix B2). The raw output was used to calculate the detection rate per 1,000 screened women given by N(DCIS)/N(mam)*1,000, with N(DCIS) number of DCIS, N(mam) number of mammograms. To calculate the observed detection rate from the number of DCIS obtained from NCR data, the number of screened women per age group was obtained from the National Evaluation Team for Breast cancer (2). Output was stratified by age group (50-54, 55-59, 60-64, 65-69, 70-74 years) and by grade (1, 2, 3). To check if observed data matched simulated data, the 95% confidence intervals (95%CI) of the simulated detection rates were compared to observed detection rates. The 95%CIs were determined with probabilistic sensitivity analysis and univariate sensitivity analyses.

A.1.2. Validation – Internal - Input

**Parameter**			**Base value**			**Reference**
**The Netherlands**		Age				
Transition	All-cause death probability (*10^{-2} / year)	0	0.324			(3)
		1-9	0.050 – 0.008			
		10-19	0.008 – 0.018			
		20-29	0.019 – 0.027			
		30-39	0.032 – 0.064			
		40-49	0.070 – 0.201			
		50-59	0.233 – 0.496			
		60-69	0.538 – 1.137			
		70-79	1.210 – 3.441			
		80-89	3.972 – 12.55			
		90-99	14.33 – 32.30			
		100	100.0			
	DCIS onset (*10^{-3} / year)	Age	*Grade 1*	*Grade 2*	*Grade 3*	(1,4)
		0-19	0.0000	0.0000	0.0000	
		20-24	0.0016	0.0003	0.0013	
		25-29	0.0019	0.0043	0.0075	
		30-34	0.0017	0.0127	0.0199	
		35-39	0.0119	0.0221	0.0406	
		40-44	0.0213	0.0516	0.0526	
		45-48	0.0398	0.0600	0.0665	
		49-54	0.2095	0.3105	0.3250	
		55-59	0.0859	0.1805	0.2558	
		60-64	0.0948	0.2396	0.3002	
		65-69	0.1016	0.2674	0.2857	
		70-75	0.1540	0.3556	0.3703	
		76-79	0.0307	0.0630	0.0491	
		80-95	0.0361	0.0585	0.0452	
		95+	0.0000	0.0000	0.0000	
	DCIS regression (/year)	20+	0.0488	0.0488	0.0488	(4–6)
	Progression to IBC (/year)	0-19	0.000	0.000	0.000	(4,7)
		20-54	0.087	0.137	0.159	
		55+	0.073	0.115	0.134	
Screening	Mammographic sensitivity		86%			(8)
	Screening frequency		Biennial			(9)
	Screening age		50-74 years			
	Participation rate		76%			
Clinical detection			5%			(10)

Appendix A.1.2 – Input parameters for SimDCIS for the Dutch population. DCIS = ductal carcinoma in situ, IBC = invasive breast cancer.

A.2. Validation - External

A.2.1. Validation – External - Description

External validation was done to evaluate the performance of the model in a different screening setting. For this, observed National Health Service (NHS) data of screening in the United Kingdom (UK) in 2021 was compared to simulated output of SimDCIS (11). For this simulation, UK screening setting was simulated. In order to simulate UK screening, death probability and screening frequency, age, and participation were adjusted (Appendix A2.2). Death probability was based on data from 2021 per age group (12). Participation rate was averaged for 2010 up to and including 2019 for women aged 50-70 years (11). Transition probability of DCIS onset, regression, and progression were assumed similar for the UK and Dutch population. Raw output was transformed to detection rate per 1,000 screened women. Both number of DCIS and number of mammograms in the UK were available from NHS data, and were used to calculate detection rate per 1,000 screened women (11). Output was stratified by age groups (50-54, 55-59, 60-64, 65-69, 70-74 years). To check if observed data matched simulated data, the 95% confidence intervals (95%CI) of the simulated detection rates were compared to observed detection rates. The simulated 95%CIs were determined with univariate sensitivity analyses (Appendix A4).

A.2.2. Validation - Input - External

**Parameter**			**Base value**	**Reference**
**The United Kingdom**		Age ↓		
Transition	All-cause death probability (*10^{-2} / year)	0	0.340	(12)
		1-9	0.230 – 0.010	
		10-19	0.010 – 0.020	
		20-29	0.020 – 0.038	
		30-39	0.042 – 0.096	
		40-49	0.104 – 0.226	
		50-59	0.244 – 0.512	
		60-69	0.558 – 1.246	
		70-79	1.364 – 3.698	
		80-89	4.172 – 12.36	
		90-99	13.80 – 84.20	
		100	100.0	
Mammography	Screening frequency		Triennial	(11)
	Screening age		50-71 years	
	Participation rate		72% (65% - 80%)	

Appendix A.2.2 – Adjusted input parameters for SimDCIS for the United Kingdom compared to the Dutch screening setting.

A.3. Validation - Cross

A.3.1. Validation – Cross – Description

Cross validation was done to evaluate performance of the model compared to another existing model. For cross validation, SimDCIS output was compared to output from the MISCAN-Fadia model and to observed data from the UK Frequency Trial (13). To simulate UK screening setting, UK death probability was used (Appendix A3.2). Transition probabilities of DCIS onset, regression, and progression were assumed independent of the population. The UK Frequency trial invited women of 50 to 62 years old to participate in screening. Both control and study arms of the UK frequency trial were simulated (Appendix A3.2). The study arm screened women at baseline and three subsequent screens with an annual interval, with participation rates of 78%, 78% and 81%, respectively. The control arm screened women at baseline and one time with a triennial interval with a participation rate of 85%. Only women at baseline were included in analyses, so a baseline participation rate of 100% was used in the simulation. If women were above 50 at baseline, they had the opportunity to participate in the population-based screening program. Therefore, regular screening was also simulated for these women before trial started, with a participation rate of 75% (14). Results were averaged for age groups 53-56, 56-59, and 59-62 years. Total detected DCIS was calculated by adding the number of screen- and clinically detected DCIS and divide by the mammogram factor to adjust for the actual number of mammograms. The mammogram factor was calculated by dividing the simulated number of mammograms by the observed number. To check if observed data matched simulated data, 95% confidence intervals (95%CI) of the simulated detection rates were compared to observed detection rates. The simulated 95%CIs were determined from variation in 10 iterations.

A.3.2. Validation – Cross - Input

Parameter			Base value	Reference
**The United Kingdom**		Age ↓		
Transition	All-cause death probability (*10^{-2} / year)	0	0.340	(12)
		1-9	0.230 – 0.010	
		10-19	0.010 – 0.020	
		20-29	0.020 – 0.038	
		30-39	0.042 – 0.096	
		40-49	0.104 – 0.226	
		50-59	0.244 – 0.512	
		60-69	0.558 – 1.246	
		70-79	1.364 – 3.698	
		80-89	4.172 – 12.36	
		90-99	13.80 – 84.20	
		100	100.0	
Mammography - Control	Screening frequency		Triennial	(13)
	Screening age		50-62 years	
	Participation rate, screening round	previous	75%	(14)
		1	100%	(13)
		2	85%	
Mammography - Study	Screening frequency		Annual	(13)
	Screening age		50-62 years	
	Participation rate, screening round	previous	75%	(14)
		1	100%	(13)
		2	78%	
		3	78%	
		4	81%	

Appendix A.3.2 – Adjusted input parameters for SimDCIS for the United Kingdom compared to the Dutch screening setting.

A.4. Univariate sensitivity analyses

A.4.1. Univariate sensitivity analyses - Input

**Parameter**		**Base case (95% CI)**		
	Age	*Grade 1*	*Grade 2*	*Grade 3*
DCIS onset ($*10^{-3}$ /year)	0-19	0.0000 (0.0000 - 0.0001)	0.0000 (0.0000 - 0.0000)	0.0000 (0.0000 - 0.0000)
	20-24	0.0016 (0.0014 - 0.0018)	0.0003 (0.0002 - 0.0003)	0.0013 (0.0012 - 0.0015)
	25-29	0.0019 (0.0017 - 0.0021)	0.0043 (0.0038 - 0.0047)	0.0075 (0.0067 - 0.0082)
	30-34	0.0017 (0.0015 - 0.0018)	0.0127 (0.0114 - 0.0140)	0.0199 (0.0179 - 0.0219)
	35-39	0.0119 (0.0107 - 0.0131)	0.0221 (0.0199 - 0.0243)	0.0406 (0.0365 - 0.0447)
	40-44	0.0213 (0.0191 - 0.0234)	0.0516 (0.0465 - 0.0568)	0.0526 (0.0474 - 0.0579)
	45-48	0.0398 (0.0358 - 0.0438)	0.0600 (0.0540 - 0.0661)	0.0665 (0.0598 - 0.0731)
	49-54	0.2095 (0.1885 - 0.2304)	0.3105 (0.2795 - 0.3416)	0.3250 (0.2925 - 0.3575)
	55-59	0.0859 (0.0773 - 0.0945)	0.1805 (0.1624 - 0.1985)	0.2558 (0.2302 - 0.2813)
	60-64	0.0948 (0.0853 - 0.1043)	0.2396 (0.2156 - 0.2635)	0.3002 (0.2702 - 0.3302)
	65-69	0.1016 (0.0915 - 0.1118)	0.2674 (0.2407 - 0.2942)	0.2857 (0.2571 - 0.3143)
	70-75	0.1540 (0.1386 - 0.1694)	0.3556 (0.3201 - 0.3912)	0.3703 (0.3332 - 0.4073)
	76-79	0.0307 (0.0277 - 0.0338)	0.0630 (0.0567 - 0.0693)	0.0491 (0.0442 - 0.0540)
	80-95	0.0361 (0.0324 - 0.0397)	0.0585 (0.0527 - 0.0644)	0.0452 (0.0407 - 0.0497)
	95+	0.0000 (0.0000 - 0.0013)	0.0000 (0.0000 - 0.0013)	0.0000 (0.0000 - 0.0026)
DCIS regression (/year)	20+	0.0488 (0.000 - 0.0952)	0.0488 (0.000 - 0.0952)	0.0488 (0.000 - 0.0952)
Progression to IBC (/year)	0-19	0.000 (0.000 - 0.000)	0.000 (0.000 - 0.000)	0.000 (0.000 - 0.000)
	20-54	0.087 (0.066 - 0.112)	0.137 (0.104 - 0.176)	0.159 (0.121 - 0.205)
	55+	0.073 (0.056 - 0.093)	0.115 (0.088 - 0.146)	0.134 (0.102 - 0.170)
Sensitivity		86% (83% - 89%)		
Clinical detection		5% (0% - 10%)		
Participation rate		76% (NL: 70% - 80%; UK: 65-80%)		

Appendix A.4.1 – Input parameters for the univariate sensitivity analyses with 95% confidence intervals (95%CI) of SimDCIS in the Dutch population. For each parameter, the lower and upper 95%CI were individually changed. DCIS = ductal carcinoma in situ, IBC = invasive breast cancer.

A.4.2. Univariate sensitivity analyses

A.4.2.1 – USA plot: screen-detected DCIS total

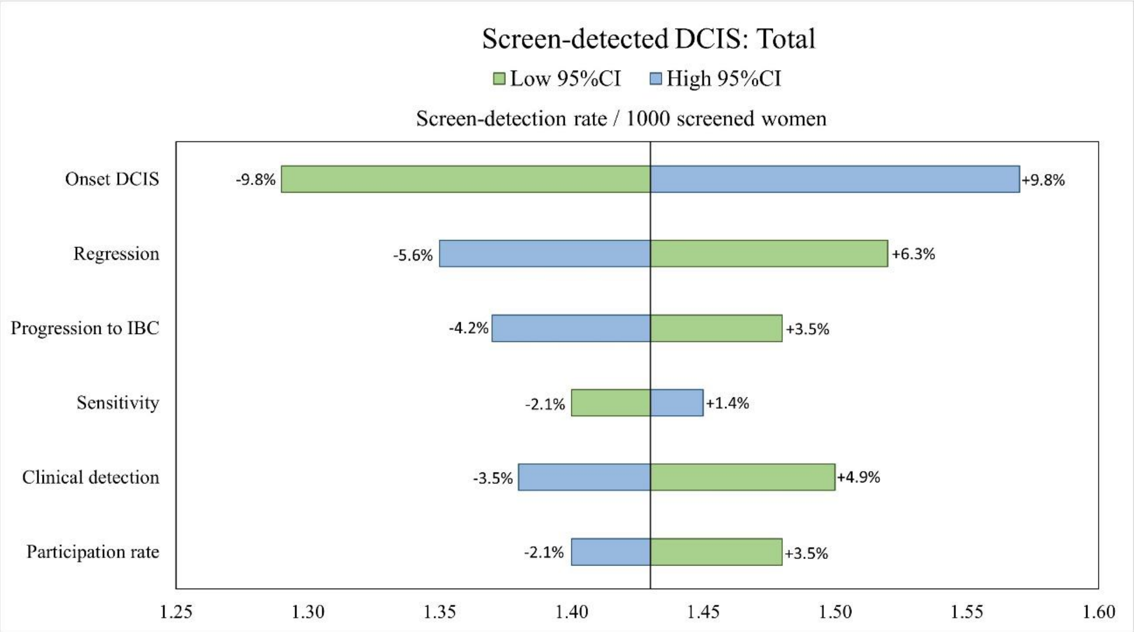

Appendix A.4.2.1 – Tornado plot of the univariate sensitivity analyses and the resulting variation in screen-detection rate per 1000 screened women in Dutch screening setting.

A.4.2.2 – USA plot: screen-detected DCIS stratified by grade

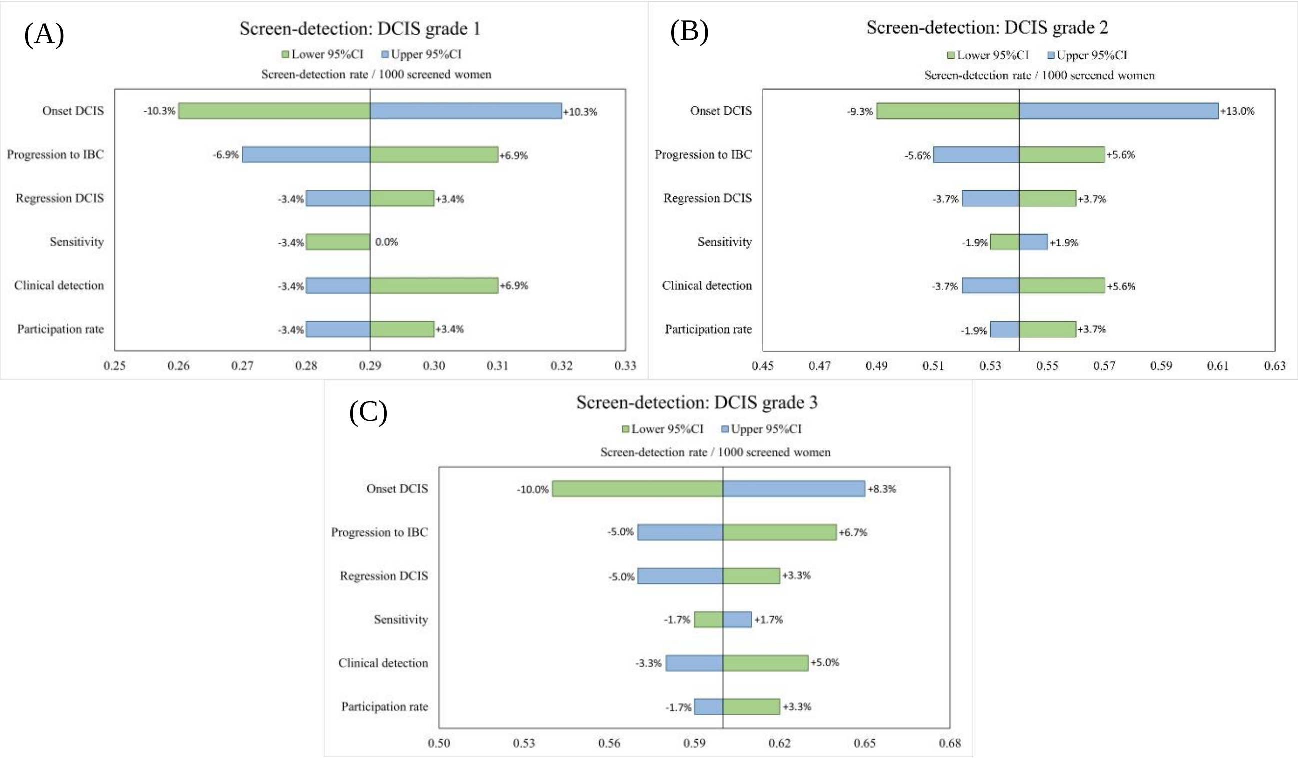

Appendix A.4.2.2 – Tornado plot of the univariate sensitivity analyses and the resulting variation in screen-detection rate per 1000 screened women of DCIS grade 1 (A), 2 (B), and 3 (C) in Dutch screening setting.

A.4.2.3 – USA plot: screen-detected DCIS stratified by age group

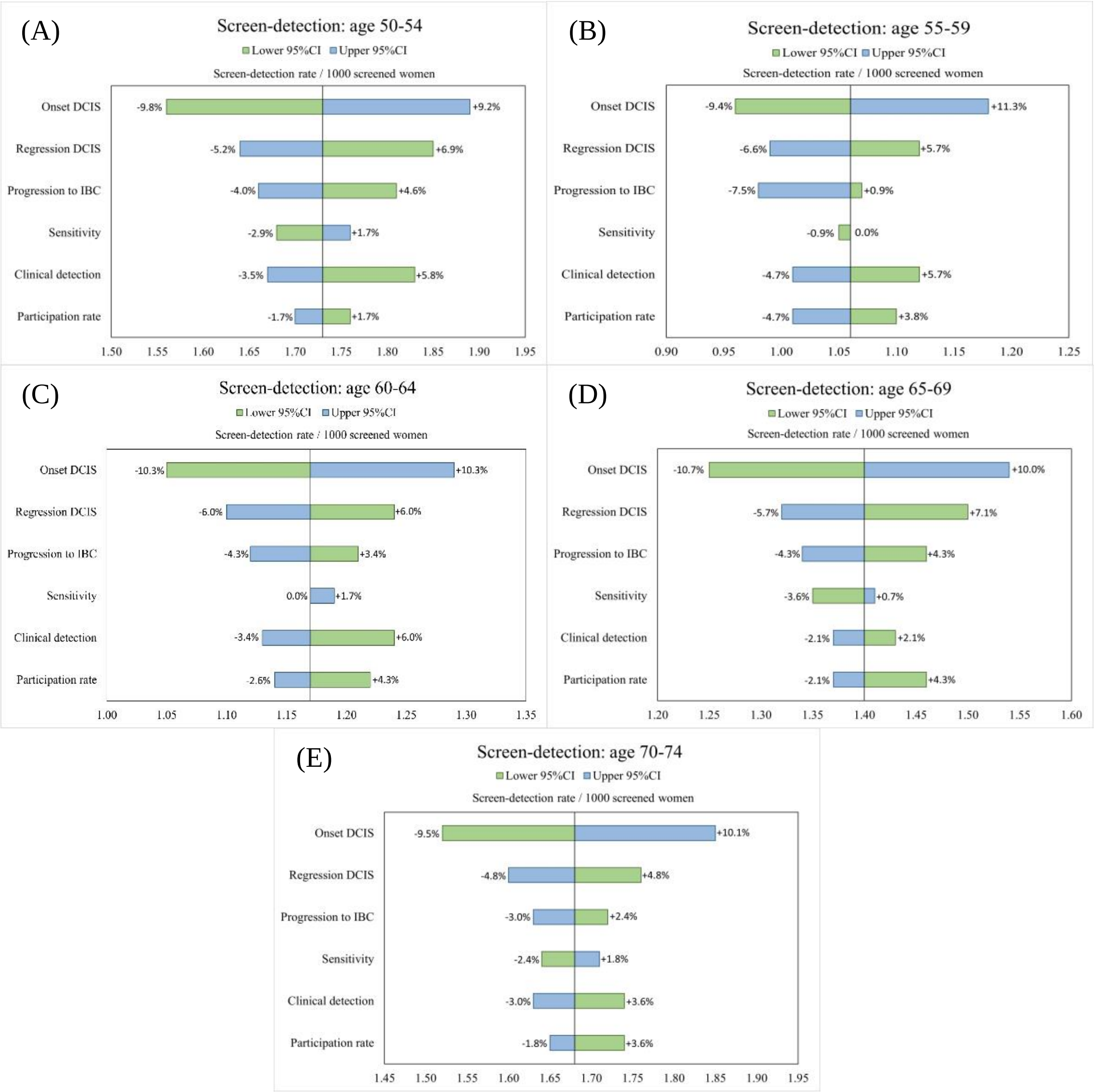

Appendix A.4.2.3 – Tornado plot of the univariate sensitivity analyses and the resulting variation in screen-detection rate per 1000 screened women in women aged 50-54 (A), 55-59 (B), 60-64 (C), 65-69 (D), 70-74 (E) in Dutch screening setting.

A.5. Probabilistic sensitivity analysis

A.5.1. Probabilistic sensitivity analysis – Input

**Parameter**		**Average (range)**		
	Age	*Grade 1*	*Grade 2*	*Grade 3*
DCIS onset ($*10^{-3}$ /year)	0-19	0.0000 (0.0000 - 0.0001)	0.0000 (0.0000 - 0.0000)	0.0000 (0.0000 - 0.0000)
	20-24	0.0016 (0.0015 - 0.0018)	0.0003 (0.0002 - 0.0003)	0.0013 (0.0012 - 0.0015)
	25-29	0.0019 (0.0017 - 0.0021)	0.0043 (0.0039 - 0.0047)	0.0075 (0.0068 - 0.0081)
	30-34	0.0017 (0.0015 - 0.0018)	0.0127 (0.0115 - 0.0137)	0.0199 (0.0179 - 0.0217)
	35-39	0.0119 (0.0109 - 0.0130)	0.0222 (0.0201 - 0.0242)	0.0407 (0.0376 - 0.0442)
	40-44	0.0214 (0.0193 - 0.0233)	0.0520 (0.0474 - 0.0564)	0.0527 (0.0477 - 0.0573)
	45-48	0.0400 (0.0363 - 0.0435)	0.0594 (0.0545 - 0.0659)	0.0664 (0.0602 - 0.0730)
	49-54	0.2090 (0.1892 - 0.2292)	0.3101 (0.2824 - 0.3369)	0.3250 (0.2937 - 0.3552)
	55-59	0.0860 (0.0783 - 0.0930)	0.1807 (0.1634 - 0.1962)	0.2551 (0.2317 - 0.2773)
	60-64	0.0952 (0.0865 - 0.1034)	0.2415 (0.2194 - 0.2612)	0.3003 (0.2737 - 0.3275)
	65-69	0.1015 (0.0926 - 0.1103)	0.2680 (0.2442 - 0.2933)	0.2875 (0.2618 - 0.3120)
	70-75	0.1536 (0.1395 - 0.1658)	0.3563 (0.3271 - 0.3886)	0.3696 (0.3393 - 0.4042)
	76-79	0.0308 (0.0279 - 0.0336)	0.0630 (0.0575 - 0.0686)	0.0491 (0.0447 - 0.0538)
	80-95	0.0361 (0.0328 - 0.0395)	0.0589 (0.0532 - 0.0642)	0.0451 (0.0411 - 0.0494)
	95+	0.0000 (0.0000 - 0.0000)	0.0000 (0.0000 - 0.0000)	0.0000 (0.0000 - 0.0000)
DCIS regression (/year)	20+	0.0477 (0.0060 - 0.0851)	0.0486 (0.0060 - 0.0921)	0.0486 (0.0060 - 0.0921)
Progression to IBC (/year)	0-19	0.000 (0.000 - 0.000)	0.000 (0.000 - 0.000)	0.000 (0.000 - 0.000)
	20-54	0.088 (0.067 - 0.111)	0.138 (0.112 - 0.172)	0.162 (0.125 - 0.203)
	55+	0.076 (0.058 - 0.090)	0.119 (0.090 - 0.144)	0.137 (0.109 - 0.166)
Sensitivity		86% (83% - 88%)		
Clinical detection		5% (1% - 9%)		
Participation rate		75% (71% - 80%)		

Appendix A.5.1 – Average and range of input parameters for 100 simulations of the probabilistic sensitivity analysis of SimDCIS in the Dutch population after random sampling from an inverse beta distribution of the lower and upper 95% confidence intervals with an alpha and beta of 2. DCIS = ductal carcinoma in situ, IBC = invasive breast cancer.

A.5.2. Probabilistic sensitivity analyses – Output

A.5.2.1 – PSA plot: screen-detected DCIS total

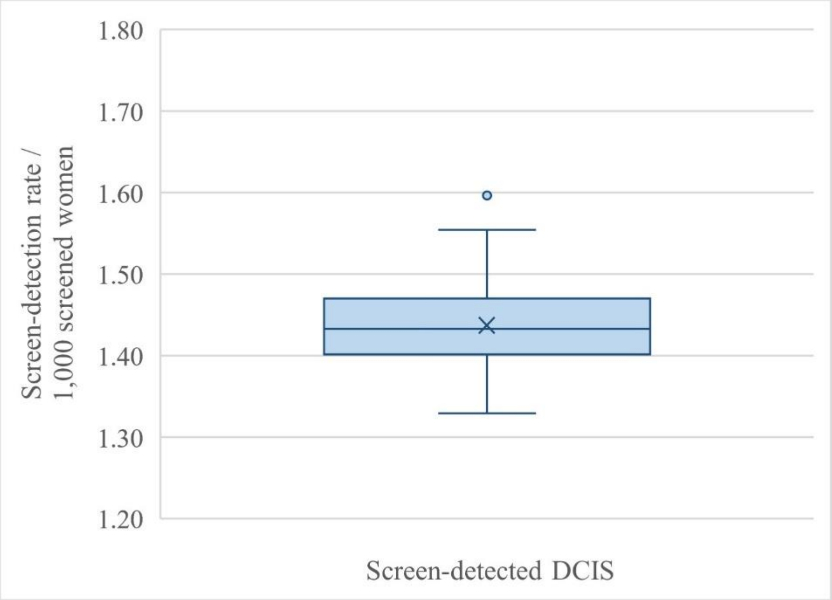

Appendix A.5.2.1 – Box-and-whisker plot of the probabilistic sensitivity analyses and the resulting variation in screen-detection rate per 1,000 screened women.

A.5.2.2 – PSA plot: screen-detected DCIS stratified by grade

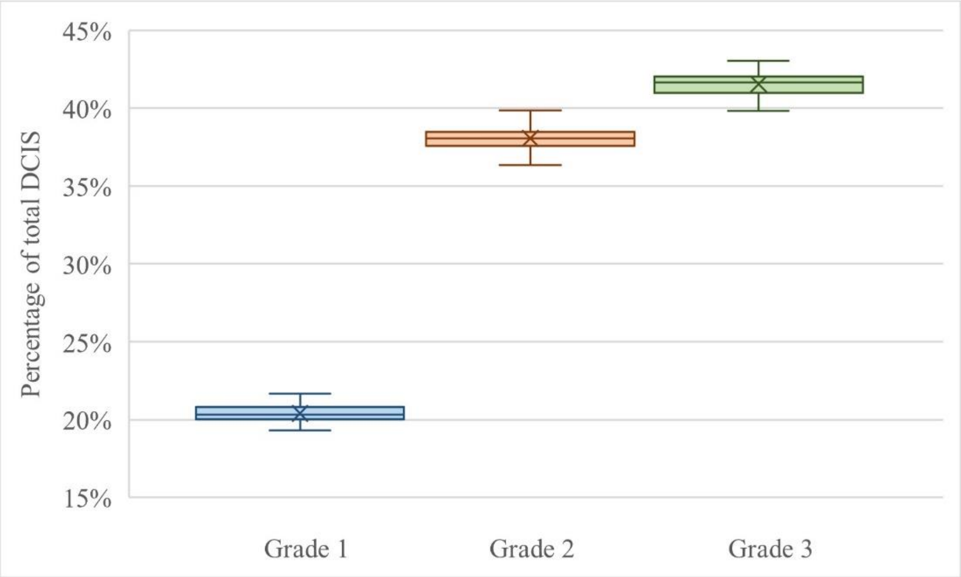

Appendix A.5.2.2 – Box-and-whisker plot of the probabilistic sensitivity analyses and the resulting variation in screen-detection rate per 1000 screened women stratified by grade for DCIS grade 1, 2, and 3.

A.6. Overview scenarios output

A.6.1 – Percentiles output all scenarios

	Regress	Progress to IBC	Clinically detected	Screen-detected	Grade distribution* Screen-detected DCIS		
					1	2	3
**Validation**							
**NL**	8%	19%	8%	61%	20%	38%	42%
**UK**	12%	27%	12%	44%	21%	38%	41%
**Univariate sensitivity analyses**							
**mean**	8%	20%	8%	61%	20%	38%	42%
**min**	0%	16%	0%	58%	20%	38%	41%
**5%**	4%	17%	4%	58%	20%	38%	41%
**25%**	7%	18%	7%	59%	20%	38%	42%
**50%**	8%	19%	8%	61%	20%	38%	42%
**75%**	8%	21%	8%	63%	20%	38%	42%
**95%**	11%	23%	11%	64%	21%	38%	42%
**max**	14%	24%	13%	65%	21%	39%	42%
**Probabilistic sensitivity analysis**							
**mean**	8%	20%	8%	61%	20%	38%	42%
**min**	1%	17%	2%	56%	19%	36%	39%
**5%**	2%	18%	3%	57%	20%	37%	40%
**25%**	5%	19%	7%	59%	20%	38%	41%
**50%**	8%	20%	8%	61%	20%	38%	42%
**75%**	10%	21%	9%	62%	21%	38%	42%
**95%**	12%	23%	12%	64%	21%	39%	43%
**max**	13%	24%	13%	65%	22%	40%	44%

*Appendix A.6.1 – Overview of DCIS estimates for validation, and min, 5th, 25th, 50th, 75th, 95th, and max percentiles for univariate and probabilistic sensitivity analyses of the Dutch screening setting. All percentages are rounded. min = minimum, max = maximum.*DCIS = ductal carcinoma in situ, IBC = invasive breast cancer.*

Appendix B – SimDCIS

B.1. Calculation transition probabilities

B.1.1. P1 - Death probability

Death probability (P1) was used in the transition of healthy to death and of DCIS to death. Death probability was calculated from Dutch mortality data of 2011 from the Central Bureau of Statistics (3). The death probability per year of age was calculated from the number of women who died and were alive per year of age in 2011. This resulted in an age-dependent yearly death probability. To ensure all women leave the model, death probability at 100 years was set to 1.0. The death probability is population-dependent and should be adjusted when SimDCIS is used in other populations.

B.1.2. P2 - DCIS onset probability

DCIS onset probability (P2) was used in the transition of healthy to DCIS. To calculate DCIS onset probability, data from the Netherlands Cancer Registry (NCR) was used (1). Data on all DCIS (screen- and not screen-detected) per grade (1, 2, 3, unknown) and 5-year age group from 2015 up to and including2022 was requested. Data from 2020 was excluded because of variations in data due to the COVID-19 pandemic. Unknown grade was also excluded. DCIS onset rate was calculated by the average number of DCIS per 5-year age group and grade (1, 2, 3) divided by the number of women alive in the Netherlands in 2018 (3). DCIS onset rate r was transformed to DCIS onset probability p using the following formula (4):

$p = 1-\exp(-rt)$ (Formula 1)

with t = 1 for a yearly transition rate.

To obtain the final DCIS onset probability, a few assumptions were made. Firstly, Dutch DCIS data had some deviations in 5-year age groups, and these groups were used as data was provided: 0-19, 45-48, 49-54, 70-75, 76-79, 95+ years. Secondly, the number of women alive in 2018 was only available for 10-year age groups. Therefore, the assumption was that the number of women per 10-year age group was equally divided to obtain 5-year age groups. Thirdly, the oldest age group with known number of women alive was 80+. Therefore, DCIS of 80-94 was summed and divided by the 80+ women, and probability of women 95+ years old was set to 0.

B.1.3. P3 – DCIS regression probability

DCIS regression probability (P3) was used in the transition of DCIS to healthy. Regression from invasive breast cancer (IBC) to DCIS was assumed not possible. As the natural history of DCIS is currently unobservable, the true number of DCIS that regress is unknown. A regression rate of 5% per year was chosen based on regression estimates ranging from 1-10% (5), and a previous modelling study (10). This 5% was adjusted to a transition probability with the previous formula (Appendix B1.2). Regression probability was not adjusted for age and grade to avoid unnecessary assumptions.

B.1.4. P4 – DCIS progression to IBC probability

DCIS progression to IBC probability (P4) was used in the transition of DCIS to IBC. Data from the US National Cancer Institute's Surveillance, Epidemiology, and End Results program of 1992–2014 contained 10-year probabilities of progression for age groups <55, and >55 (7). The probabilities were first calculated to yearly rates using Formula 1, with t = 10 (4). The probability for 0-19 year old women was set to 0, in line with DCIS onset being 0 for this age group. The yearly age-dependent rates were transformed back to transition probabilities using Formula 1, where again t was set to 10 (Appendix B1.2). This resulted in an age-dependent progression probability. To also account for the difference in progression probability per grade, a grade dependency factor was calculated and used to calculate grade-dependent probabilities. The average grade dependency within age groups was calculated, and normalized to have an average of 1.0 over all estimates. This resulted in a grade dependency factor of 0.68, 1.07, and 1.24 for DCIS grade 1, 2, and 3, respectively. The grade dependency factor was multiplied with the calculated age-dependent progression probability, resulting in the final age- and grade-dependent progression probability.

B.2. Model output

B.2.1. Model output - Codebook

Code	State	DCIS grade	Description
00	Healthy	None	Healthy, no tumour present
01		Previous 1	Healthy, previous grade 1 DCIS that regressed
02		Previous 2	Healthy, previous grade 2 DCIS that regressed
03		Previous 3	Healthy, previous grade 3 DCIS that regressed
10	Death	None	Death, no tumour present
11		1	Natural death with grade 1 DCIS present
12		2	Natural death with grade 2 DCIS present
13		3	Natural death with grade 3 DCIS present
21	DCIS	1	Grade 1 DCIS present
22		2	Grade 2 DCIS present
23		3	Grade 3 DCIS present
31	IBC	1	Grade 1 IBC present
32		2	Grade 2 IBC present
33		3	Grade 3 IBC present
41	Screen-detected	1	Grade 1 DCIS, detected in population-based screening
42		2	Grade 2 DCIS, detected in population-based screening
43		3	Grade 3 DCIS, detected in population-based screening
51	Clinically detected	1	Grade 1 DCIS, detected outside of screening
52		2	Grade 2 DCIS, detected outside of screening
53		3	Grade 3 DCIS, detected outside of screening

Appendix B.2.1. – Code with definitions of the raw output of SimDCIS. DCIS = ductal carcinoma in situ, IBC = invasive breast cancer.

B.2.2. Model output – Example output

**Raw output**										
40	*00*	*00*	*10*							
77	*00*	*00*	*00*	*00*	*00*	*00*	*00*	*00*	*00*	*00*
	00	*00*	*00*	*00*	*00*	*00*	*00*	*00*	*00*	*00*
	00	*00*	*00*	*00*	*00*	*00*	*00*	*00*	*00*	*00*
	00	*00*	*00*	*00*	*00*	*00*	*00*	*00*	*00*	*00*
	00	*00*	*00*	*00*	*00*	*00*	*00*	*00*	*00*	*00*
	00	*00*	*00*	*00*	*00*	*00*	*00*	*00*	*00*	*00*
	00	*22*	*22*	*22*	*22*	*22*	*22*	*22*	*42*	
185	*00*	*00*	*00*	*00*	*00*	*00*	*00*	*00*	*00*	*00*
	00	*00*	*00*	*00*	*00*	*00*	*00*	*00*	*00*	*00*
	00	*00*	*00*	*00*	*00*	*00*	*00*	*00*	*00*	*00*
	00	*00*	*00*	*00*	*00*	*00*	*00*	*00*	*00*	*00*
	00	*00*	*00*	*00*	*00*	*00*	*22*	*32*		
206	*00*	*00*	*00*	*00*	*00*	*00*	*00*	*00*	*00*	*00*
	00	*00*	*00*	*00*	*00*	*00*	*00*	*00*	*00*	*00*
	00	*00*	*00*	*00*	*00*	*00*	*00*	*00*	*00*	*00*
	00	*00*	*00*	*00*	*00*	*00*	*00*	*00*	*00*	*00*
	00	*00*	*00*	*00*	*00*	*00*	*00*	*00*	*00*	*00*
	00	*00*	*00*	*00*	*00*	*00*	*00*	*00*	*00*	*00*
	00	*00*	*00*	*00*	*00*	*00*	*00*	*00*	*00*	*00*
	00	*00*	*00*	*00*	*23*	*53*				
1074	*00*	*00*	*00*	*00*	*00*	*00*	*00*	*00*	*00*	*00*
	00	*00*	*00*	*00*	*00*	*00*	*00*	*00*	*00*	*00*
	00	*00*	*00*	*00*	*00*	*00*	*00*	*00*	*00*	*00*
	00	*00*	*00*	*00*	*00*	*00*	*00*	*00*	*00*	*00*
	00	*00*	*00*	*00*	*00*	*00*	*00*	*00*	*00*	*00*
	00	*00*	*00*	*00*	*23*	*03*	*03*	*03*	*03*	*03*
	03	*03*	*03*	*03*	*03*	*03*	*03*	*03*	*03*	*03*
	03	*03*	*03*	*03*	*03*	*03*	*03*	*03*	*03*	*03*
	03	*03*	*03*	*03*	*03*	*03*	*13*			

Appendix B.2.2. – Raw output of SimDCIS with the first number counting the women and the following numbers indicating the state and grade of this woman per year. Examples given: Woman 40 dies at age 2. Woman 77 develops DCIS grade 2 at age 61 and is screen-detected at age 68. Woman 185 develops DCIS grade 2 at 46 that progresses to IBC at 47. Woman 206 develops DCIS grade 3 at age 74 and is clinically detected at age 75. Woman 1074 develops DCIS grade 3 at age 54 that regresses at age 55 after which she dies at age 86.

References

1. IKNL. iknl.nl/nkr-cijfers. NKR cijfers borstkanker'.
2. Landelijk Evaluatie Team voor bevolkingsonderzoek naar Borstkanker (LETB). Landelijke evaluatie van bevolkingsonderzoek naar borstkanker in Nederland LETB XIII. 2014.
3. CBS. https://opendata.cbs.nl/#/CBS/nl/. StatLine: Nederland in cijfers.
4. Gidwani R, Russell LB. Estimating Transition Probabilities from Published Evidence: A Tutorial for Decision Modelers. Pharmacoeconomics. 2020 Nov 1;38(11):1153–64.
5. Poelhekken K, Lin Y, Greuter MJW, van der Vegt B, Dorrius M, de Bock GH. The natural history of ductal carcinoma in situ (DCIS) in simulation models: A systematic review. Vol. 71, Breast. Churchill Livingstone; 2023. p. 74–81.
6. Segnan N, Minozzi S, Armaroli P, Cinquini M, Bellisario C, González-Lorenzo M, et al. Epidemiologic evidence of slow growing, nonprogressive or regressive breast cancer: A systematic review. Vol. 139, International Journal of Cancer. Wiley-Liss Inc.; 2016. p. 554–73.
7. Ryser MD, Weaver DL, Zhao F, Worni M, Grimm LJ, Gulati R, et al. Cancer Outcomes in DCIS Patients Without Locoregional Treatment. J Natl Cancer Inst. 2019 Sep 1;111(9):952–60.
8. Ernster VL, Ballard-Barbash R, Barlow WE, Zheng Y, Weaver DL, Cutter G, et al. Detection of Ductal Carcinoma In Situ in Women Undergoing Screening Mammography. J Natl Cancer Inst. 2002;94(20).
9. IKNL. Monitor bevolkingsonderzoek borstkanker 2020-2021. 2021;
10. van Luijt PA, Heijnsdijk EAM, Fracheboud J, Overbeek LIH, Broeders MJM, Wesseling J, et al. The distribution of ductal carcinoma in situ (DCIS) grade in 4232 women and its impact on overdiagnosis in breast cancer screening. Breast Cancer Research. 2016 May 10;18(1).
11. NHS. Breast Screening Programme, England 2019-20. 2021.
12. Statista. Age-specific death rate per 1,000 population in the United Kingdom in 2021 by gender. 2022.
13. Van Ravesteyn NT, Heijnsdijk EAM, Draisma G, De Koning HJ. Prediction of higher mortality reduction for the UK Breast Screening Frequency Trial: A model-based approach on screening intervals. Br J Cancer. 2011 Sep 27;105(7):1082–8.
14. Sasieni P. Evaluation of the UK breast screening programmes. Annals of Oncology. 2003 Aug 1;14(8):1206–8.
